# Improving Discrimination in Color Vision Deficiency by Image Re-Coloring

**DOI:** 10.3390/s19102250

**Published:** 2019-05-15

**Authors:** Huei-Yung Lin, Li-Qi Chen, Min-Liang Wang

**Affiliations:** 1 Department of Electrical Engineering, Advanced Institute of Manufacturing with High-Tech Innovation, National Chung Cheng University, Chiayi 621, Taiwan; 2 Department of Electrical Engineering, National Chung Cheng University, Chiayi 621, Taiwan; kenbroms@gmail.com; 3 Asian Institute of TeleSurgery/IRCAD-Taiwan, Changhua 505, Taiwan; ccuvislab@hotmail.com

**Keywords:** color vision deficiency, image re-coloring, visual assistance

## Abstract

People with color vision deficiency (CVD) cannot observe the colorful world due to the damage of color reception nerves. In this work, we present an image enhancement approach to assist colorblind people to identify the colors they are not able to distinguish naturally. An image re-coloring algorithm based on eigenvector processing is proposed for robust color separation under color deficiency transformation. It is shown that the eigenvector of color vision deficiency is distorted by an angle in the λ, *Y-B*, *R-G* color space. The experimental results show that our approach is useful for the recognition and separation of the CVD confusing colors in natural scene images. Compared to the existing techniques, our results of natural images with CVD simulation work very well in terms of RMS, HDR-VDP-2 and an IRB-approved human test. Both the objective comparison with previous works and the subjective evaluation on human tests validate the effectiveness of the proposed method.

## 1. Introduction

Most human beings have the ability of color vision perception, which senses the frequency of the light reflected from object surfaces. However, color vision deficiency (CVD) is a common genetic condition [1]. It is in general not a fatal or serious disease, but still brings inconvenience to most patients. People with color vision deficiency (or so-called color blindness) cannot observe the colorful world due to the damage of color reception nerves. Whether caused by genetic problems or chemical injury, the damaged nerves are not able to distinguish certain colors. There are a few common types of color vision deficiency such as protanomaly (red weak), deuteranomaly (green weak) and tritanomaly (blue weak). They can be detected and verified easily by some special color patterns (e.g., Ishihara plates [2]), but, unfortunately, cannot be cured by medical surgery or other treatments. Compared to the human population, people with color vision deficiency are still a minority, and they are sometimes ignored and restricted by our society.

In many places, colorblind people are not allowed to have a driver’s license. A number of careers in engineering, medicine and other related fields have set some restrictions on the ability of color perception. The display and presentation of most media on devices and in many forms do not specifically take color vision deficiency into consideration. Although the weakness in distinguishing different colors does not obviously affect people’s learning and cognition, there is still a challenge in terms of color-related industries. In this work, we propose an approach to assist people with color vision deficiency to tell the difference among the confusing colors as much as possible. A simple yet reasonable technique, “color reprint”, is developed and used to represent the CVD-proof colors. The algorithm does not only preserve the naturalness and details of the scenes, but also possess real-time processing capability. It can, therefore, be implemented on low-cost or portable devices, and brought to everyday life.

**Figure 1:**
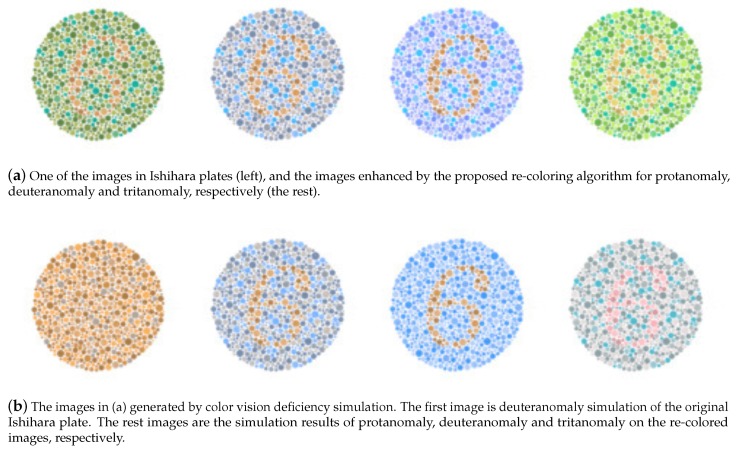
(**a**) An original image from Ishihara plates and the enhanced images using our re-coloring algorithms for protanomaly, deuteranomaly and tritanomaly. (**b**) The images generated from a color vision deficiency simulation tool [6]. The results show that our image enhancement technique is able to improve check pattern recognition under various types of color vision deficiency.

Human color vision is based on three light-sensitive pigments [3,4]. It is trichromatic and presented in three dimensions. The color stimulus is specified by the power contained at each wavelength. Normal trichromacy is because that the retina contains three classes of cone photo-pigment neural cells, L-, M-, and S-cones. A range of wavelengths of the light stimulate each of these receptor types at various degrees. For example, yellowish green light stimulates both L- and M-cones equally strongly, but S-cones weakly. Red light stimulates more L-cones than M-cones, and S-cones hardly at all. Our brain combines the information from each type of cone cells, and responds to different wavelengths of the light as shown in Table 1. The color processing is carried out in two stages. First, the stimulus from the cones is recombined to form two color-opponents and luminance. Second, an adaptive signal regulation processes within the operating range and stabilizes the illumination changes of the object appearance. When any kind of sensitive pigments is broken or loses the functionality [1], people can only view a part of the visible spectrum compared to those with normal vision capability [5] (see Figure 1).

There are studies about the molecular genetics of human color vision in the literature. Nathans et al. have described the isolation and sequencing of genomic and complementary DNA clones which encode the apoproteins of the red, green and blue pigments [4]. With newly refined methods, the number and ratio of genes are re-examined in men with normal color vision. A recent report reveals that many males have more pigment genes on the X chromosome than previously studied, and many have more than one long-wave pigment gene [7]. The loss of characteristic sensitivities of the red and green receptors introduced into the transformed sensitivity curves also indicates the appropriate degrees of luminosity deficit for deuteranopes and protanopes [8].

Color vision deficiency is mainly caused by two reasons: natural genetic factors and impaired nerves or brain. A protanope suffers from a lack of the L-cone photo-pigment, and is unable to discriminate reddish and greenish hues since the red–green opponent mechanism cannot be constructed. A deuteranope does not have sufficient M-cone photo-pigment, so the reddish and greenish hues are not distinguishable. People with tritanopia do not have the S-cone photo-pigment, and, therefore, cannot discriminate yellowish and bluish hues [9]. The literature shows that more than 8% of the world population suffer from color vision deficiency (see Table 2). For color vision correction, gene therapy which adds the missing genes is sufficient to restore full color vision without further rewiring of the brain. It has been tested on a monkey with colorblindness since birth [10]. Nevertheless, there are also non-invasive alternatives available by means of computer vision techniques.

In [12], Huang et al. propose a fast re-coloring technique to improve the accessibility for the impaired color vision. They design a method to derive an optimal mapping to maintain the contrast between each pair of the representative colors [13]. In a subsequent work, an image re-coloring algorithm for dichromats using the concept of key color priority is presented [14]. A color blindness plate (CBP) is presented by Chen et al., which is a satisfactory way to test color vision in the computer vision community [15]. The approach is adopted to demonstrate normal color vision, as well as red–green color vision deficiency. Rasche et al. propose a method to preserve the image details while reducing the gamut dimension, and seek a color to gray mapping to maintain the contrast and luminance consistency [16]. They also describe a method which allows the re-colored images to deliver the content with increased information to color-deficient viewers [17]. In [18], Lau et al. present a cluster-based approach to optimize the transformation for individual images. The idea is to preserve the information from the source space as much as possible while maintaining the natural mapping as faithfully as possible. Lee et al. develop a technique based on fuzzy logic and correction of digital images to improve the visual quality for individuals with color vision disturbance [19]. Similarly, Poret et al. design a filter based on the Ishihara color test for color blindness correction [20].

Most algorithms for color transformation aim to preserve the color information in the original image while maintaining the re-colored image as naturally as possible. This might be different from some image processing and computer vision tasks; the images appearing natural after enhancement is an important issue for color vision deficiency correction. It is not only to keep the image details intact, but also to maintain the colors as smooth as those without the re-coloring process. These conditions re-range in the color distribution space to let the colorblind people to discriminate different colors [21,22]. Moreover, it is generally agreed that color perception is subjective and will not be exactly the same for different people. In this work, the proposed method is carried out on color vision deficiency simulation tools and adopts human tests for evaluation. We use RMS (root mean squares) to calculate the change after re-coloring, and HDR-VDP (visual difference predictor) [23] to compare the visibility and quality of subjective human feeling. Our algorithms not only present the naturalness and details of the images, but process almost in real-time.

## 2. Approach

In this paper, a technique called *color warping* (CW) is proposed for effective image re-coloring. It uses the orientation of the eigenvectors of the color vision deficiency simulation results to warp the color distribution. In general, the acquired images are presented in the RGB color space for display. This is, however, not suitable for color vision-related processing. For human color perception related tasks, the images are first transformed to the λ, *Y-B*, *R-G* color space based on the CIECAM02 model [24]. It consists of a transformation from RGB to LMS [25] using

(1) $$\left[\begin{array}{c} L\\ M\\ S \end{array}\right]=\left[\begin{array}{ccc} 0.7328 & 0.4296 & -0.1624\\ -0.7036 & 1.6975 & 0.0061\\ 0.0030 & 0.0136 & 0.9834 \end{array}\right]\left[\begin{array}{c} R\\ G\\ B \end{array}\right]$$

followed by a second transformation from LMS to λ, *Y-B*, *R-G* with

(2) $$\left[\begin{array}{c} \lambda \\ Y-B\\ R-G \end{array}\right]=\left[\begin{array}{ccc} 0.6 & 0.4 & 0.0\\ 0.24 & 1.05 & -0.7\\ 1.2 & -1.6 & 0.4 \end{array}\right]\left[\begin{array}{c} L\\ M\\ S \end{array}\right].$$

Since the above transformations are linear, it is easily to verify the relationship between the RGB and λ, *Y-B*, *R-G* color spaces is given by

(3) $$\left[\begin{array}{c} \lambda \\ Y-B\\ R-G \end{array}\right]=\left[\begin{array}{ccc} 0.3479 & 0.5981 & -0.3657\\ -0.0074 & -0.1130 & -1.1858\\ 1.1851 & -1.5708 & 0.3838 \end{array}\right]\left[\begin{array}{c} R\\ G\\ B \end{array}\right]$$

and

(4) $$\left[\begin{array}{c} R\\ G\\ B \end{array}\right]=\left[\begin{array}{ccc} 1.2256 & -0.2217 & 0.4826\\ 0.9018 & -0.3645 & -0.2670\\ -0.0936 & -0.8072 & 0.0224 \end{array}\right]\left[\begin{array}{c} \lambda \\ Y-B\\ R-G \end{array}\right].$$

**Figure 2:**
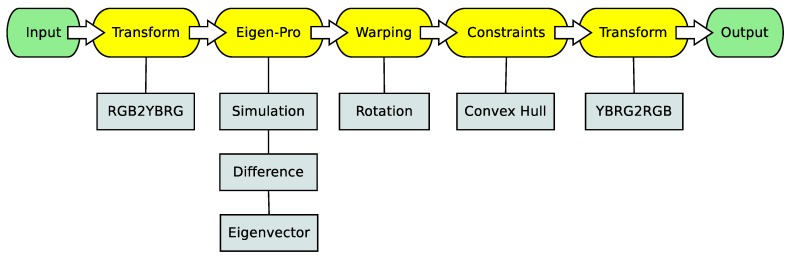
The flowchart of the proposed technique. In the pipeline, the images are first transformed to the λ, *Y-B*, *R-G* color space for the re-color processing, followed by a transformation back to the original RGB color space.

A flowchart of the proposed method is illustrated in Figure 2. The “Eigen-Pro” stage represents the eigenvector processing. The *color warping* is the key idea of this work, and the color constraints are used to make the distortion decrease after the color space transformation.

### 2.1. Color Transform

The physical property of the light used for color perception is the distribution of the spectral power [26]. In principle, there are many distinct spectral colors, and the set of all physical colors may be thought of as a large-dimensional vector space. A better alternative to the commonly adopted tristimulus coordinates for the spectral property of the light is to use L-, M-, and S-cone cells coordinates as a 3-space. To form a model for human perceptual color space, we can consider all the resulting combinations as a subset of the 3-space. The property of the cones covers the region away from the origin corresponding to the intensity of the S, M and L lights proportionately. A digital image acquisition device consists of different elements [27,28]. The characteristics of the light and the material of the observed object determine the physical properties of its color [27,29,30]. For color transformation, Huang et al. [31] present a method to warp images to the CIELab color space by rotating a matrix. Dana et al. [32] and Swain et al. [33] propose to use an antagonist space which does not take the non-linear human eye response into consideration. Instead, we transform the color space to (WS, RG, BY) based on the electro-physiological study [34].

### 2.2. Eigenvector Processing

**Figure 3:**
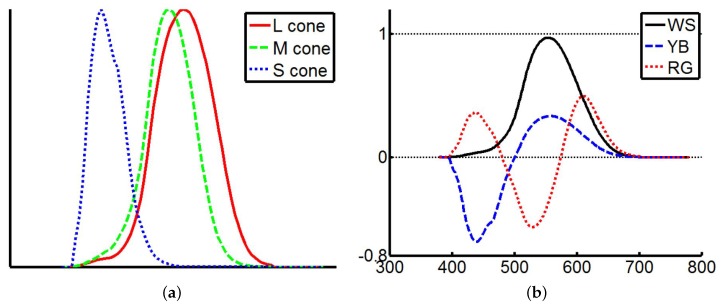
The cone spectral sensitivity functions at all wavelengths in the visible range. (**a**) Responding curve [36]. (**b**) Spectral response functions for the opponent channels [34].

Color vision deficiency cannot be understood easily by most people with normal vision. Thus, it is necessary to use simulation tools to create synthetic images for ordinary viewers to understand what are seen by the colorblind people [35]. Some well-known tools include Colblindor [6] and LMS [25], and there are also several websites to perform the simulation online (For example, Coblis Color Blindness Simulator (http://www.color-blindness.com/coblis-color-blindness-simulator), Color Blindness Simulator (http://www.etre.com/tools/colourblindsimulator), and Vision Simulator (http://www.webexhibits.org/causesofcolor/2.html)). In this work, Machado’s approach is adopted for our color vision deficiency simulation [35]. It utilizes a physiology based simulation model to achieve the sensation of cones in human visual perception. The simulation is to shift the pigments of the responding curve of spectral sensitivity functions as shown in Figure 3. Anomalous trichromacy can be simulated by shifting the sensitivity of the L, M, and S cones in the following ways:

- Protanomaly: Shift L cone toward M cone, $L{\left(\lambda \right)}_{a}=L\left(\lambda +\Delta \lambda_{L}\right)$.
- Deuteranomaly: Shift M cone toward L cone, $M{\left(\lambda \right)}_{a}=M\left(\lambda +\Delta \lambda_{M}\right)$.
- Trianomaly: Shift S cone, $S{\left(\lambda \right)}_{a}=S\left(\lambda +\Delta \lambda_{S}\right)$.

The elements of the transformation matrix Γ can be derived by

(5) $$f{\left(\lambda ,R,G,B\right)}_{WS,YB,RG}=\rho_{R,G,B}\int \phi_{R,G,B}\left(\lambda \right)f{\left(\lambda \right)}_{WS,YB,RG}\;d\lambda$$

where $\phi_{R,G,B}$ is the spectral power distribution function, and $\rho_{R,G,B}$ is a normalization factor. Thus, Γ is the projection of the spectral power distributions of RGB primaries onto a set of basic functions $f{\left(\lambda ,R,G,B\right)}_{WS,YB,RG}$. That is,

(6) $$\Gamma =\left[\begin{array}{ccc} f{\left(R\right)}_{WS} & f{\left(G\right)}_{WS} & f{\left(B\right)}_{WS}\\ f{\left(R\right)}_{YB} & f{\left(G\right)}_{YB} & f{\left(B\right)}_{YB}\\ f{\left(R\right)}_{RG} & f{\left(G\right)}_{RG} & f{\left(B\right)}_{RG} \end{array}\right].$$

This model is based on the stage theory of human color vision, and is derived from the data reported in electro-physiological study [34]. Let $\Phi_{CVD}$ be the matrix that maps RGB to the opponent-color space of normal trichromacy, then the simulation of dichromatic vision is obtained by the transformation

(7) $$\left[\begin{array}{c} R_{s}\\ G_{s}\\ B_{s} \end{array}\right]=\Phi_{CVD}\left[\begin{array}{c} R\\ G\\ B \end{array}\right].$$

By definition, an eigenvector is the non-zero vector mapped by a given linear transformation of a vector space onto a vector that is the product of the original vector multiplied by a scalar. Thus, the algorithm counts the eigenvectors of the covariance matrix from the images in *Y-B*, *R-G* of the λ, *Y-B*, *R-G* opponent color space, i.e.,

(8) $$\left[v,d\right]=eig(cov\left(I_{Y-B},I_{R-G}\right))$$

where eig is the function of eigenvalue and eigenvector, and $I_{Y-B}$ and $I_{R-G}$ are the *Y-B* and *R-G* images, respectively. On the left hand side of the equation, *d* is the generalized eigenvalue, and *v* is a 2×2 matrix since the covariance cov is a 2×2 matrix derived from a pair of n×1 images given by the covariance matrix

(9) $$cov\left(X,Y\right)=\frac{\sum_{i=1}^{n}\left(X_{i}-\bar{X}\right)\left(Y_{i}-\bar{Y}\right)}{\left(n-1\right)}$$

**Figure 4:**
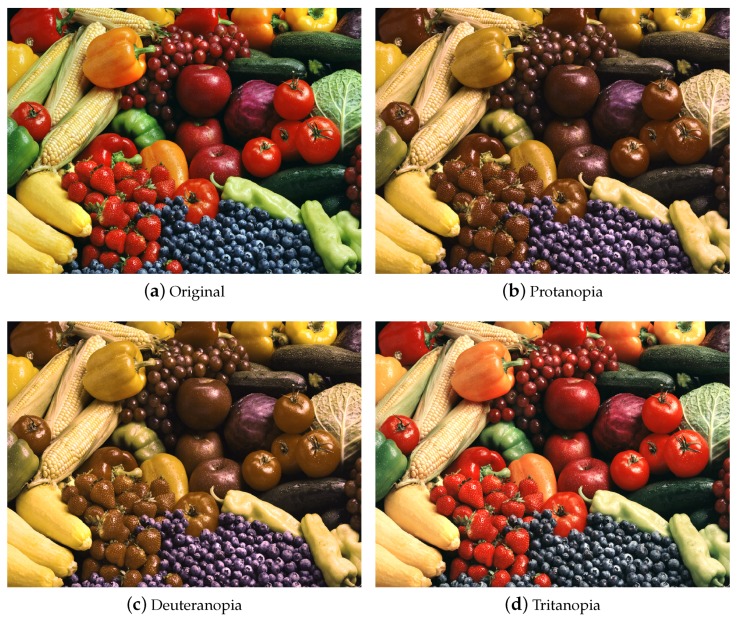
The three types of color vision deficiency simulation using Machado’s approach [35] with sensitive 0.6 and the matrix $\Phi_{CVD}$ as shown in Table 3.

**Figure 5:**
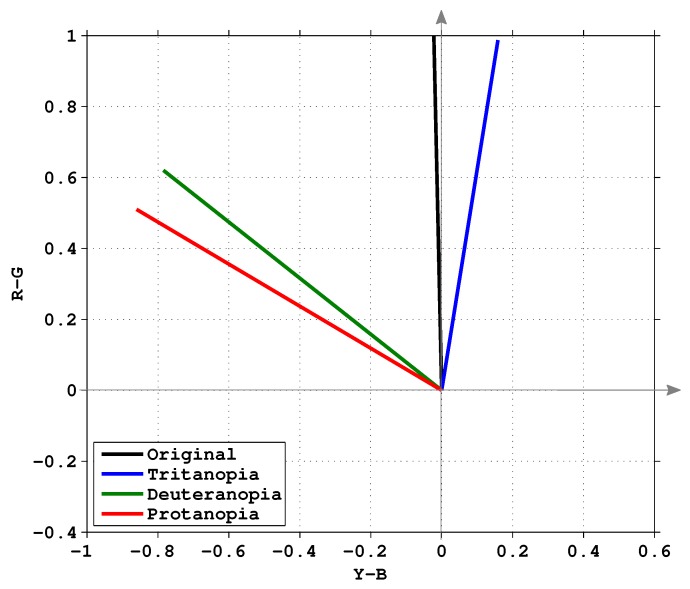
Eigenvectors of the covariance matrix of the images shown in Figure 4.

For the original and CVD simulation images shown in Figure 4, the characteristics of the associated eigenvectors are illustrated in Figure 5. The black line (at about $91^{\circ }$) indicates the eigenvector of the original image. For protanopia (red line about $150^{\circ }$) and deuteranopia (green line about $140^{\circ }$), the eigenvectors lead the one associated with the original image. The eigenvector of tritanopia (blue line at about $80^{\circ }$) is behind the original image case. Our objective is to recover the angle difference between the normal and color vision deficiency images, and use it to re-color the image. The difference image when observed by normal viewers and the color vision deficiency simulation is defined by

(10) $$I_{diff}=\sqrt{{\left(I_{n}\left(YB\right)-I_{c}\left(YB\right)\right)}^{2}+{\left(I_{n}\left(RG\right)-I_{c}\left(RG\right)\right)}^{2}}$$

where $I_{n}$ and $I_{c}$ represent the intensity observed by a normal viewer and obtained from the color vision deficiency simulation, respectively.

**Figure 6:**
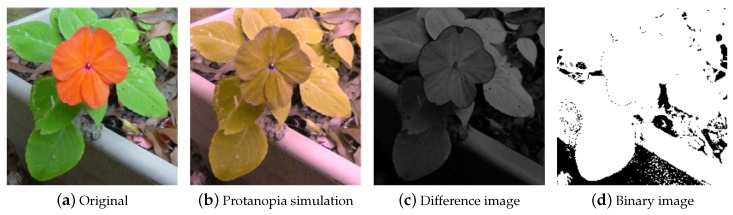
An example of Protanopia simulation. (**a**) is the original image and (**b**) is the Protanopia simulation result. (**c**) is the difference of (**a**,**b**) computed in the *λ*, *Y-B*, *R-G* color space. (**d**) is the binarized version of (**c**) for better illustration.

An example of protanopia simulation is illustrated in Figure 6. The difference image is shown in Figure 6c, and a binary image for better illustration is shown in Figure 6d. Our objective is to recover the angle and difference between the normal and CVD images and use the information to re-color the CVD simulation images.

### 2.3. Color Warping

**Figure 7:**
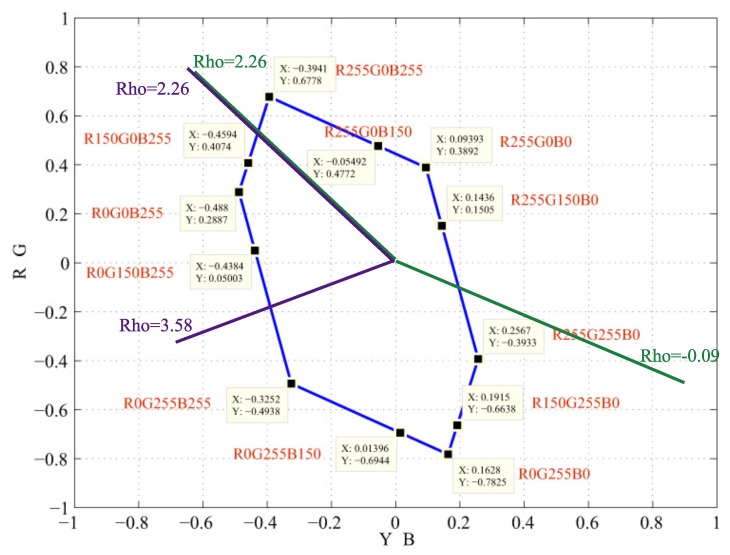
The pure colors RGB (0, 150, and 255).

**Figure 8:**
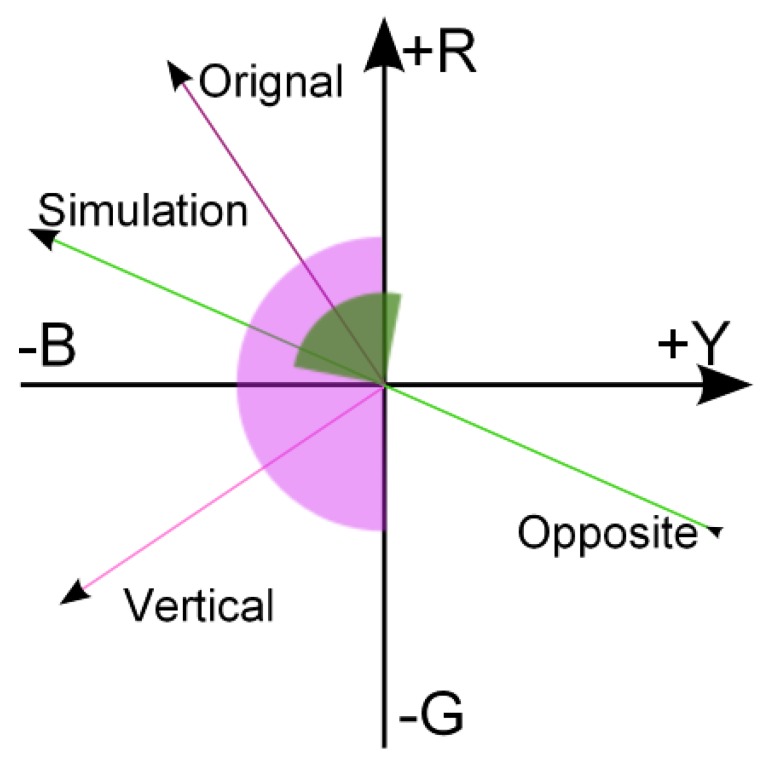
An illustration of the color warping range from the green area to the red area.

The color values of the images are transformed from RGB to the opponent color space λ, *Y-B*, *R-G* using Equations (3) and (4). It is assumed that color vision deficiency does not affected by the brightness, so the value λ corresponding to the luminance is keep intact. To define the warping range with the angle of an eigenvector, we construct twelve pure colors in RGB using the values of 0, 150 and 255. Figure 7 shows the range of missing chroma of color vision deficiency. The area within the two green lines is the red chroma missing for protanopia and deuteranopia, and the area within th two purple lines is the blue chroma missing for tritanopia. The color points represented in the Cartesian coordinates are then transformed to the polar coordinates by

(11) $$\theta =\tan^{-1}\frac{y}{x}$$

for processing. The angle θ associated with the eigenvector in the λ, *Y-B*, *R-G* color space is used to derive the range to be processed. Since the image is now in the opponent color space, the range is defined by the angle of the simulation vector to the opposite angle of the simulation vector. Finally, the warping range is defined by the vertical angle of the original vector to the opposite angle of the simulation vector. An example is illustrated in Figure 8, the green area is warped to the red area for image re-coloring.

The new color angle is derived from the original color angle by

(12) $$\theta_{new}=\frac{\theta_{⊥}-\theta_{op}}{\pi }\cdot \left(\theta -\theta_{op}\right)$$

where the angles of color points are defined in the range of [−π,π], $\theta_{⊥}$ is the angle of vector orthogonal to the original vector, and $\theta_{op}$ is the angle of vector opposite to the color vision deficiency simulation vector.

**Figure 9:**
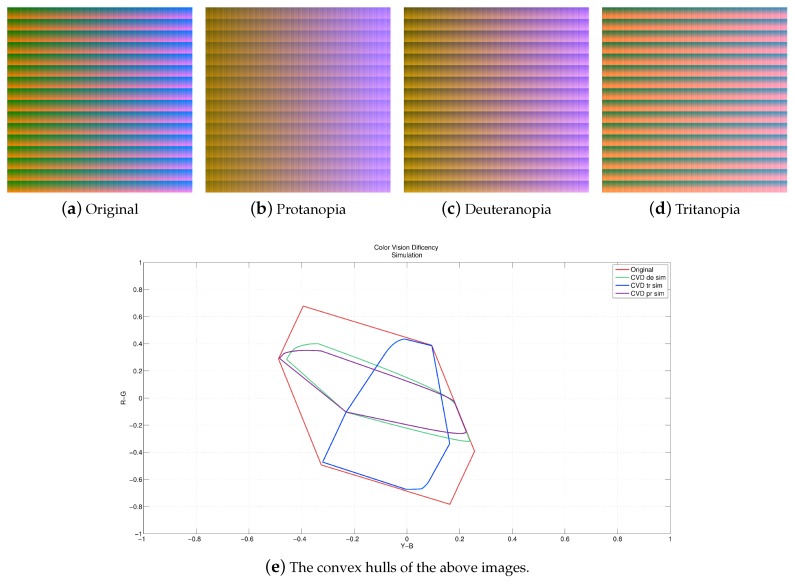
(**a**) Three types of color vision deficiency simulation using a full-color image with 256^3^ pixels. The image resolution is 4096 × 4096. (**e**) The convex hulls of the images in (**a**–**d**). All types of CVD simulation cover only a part of the convex hull of the original full color imag.

When the image is converted from RGB to the λ, *Y-B*, *R-G* color space, it is in a limited range of color space representation. We need a constraint to avoid the luminance overflow problem, which will make colors not smooth after converted back to the RGB color representation. In our approach, a convex hull is adopted for the color constraint due to its simplicity for boundary derivation. Figure 9a–d illustrate the full-color images constructed using $256^{3}$ pixels, i.e., the resolution of 4096×4096, and the corresponding convex hull is shown in Figure 9e (the red lines). The formula used for conversion is given by

(13) $$\rho_{new}=\rho \times \frac{\rho (\theta_{new})}{\rho (\theta )}$$

where ρ is the original value, $\rho (\theta_{new})$ is the value of the convex hull at $\theta_{new}$, and ρ(θ) is the value of the convex hull at θ. The resulting image in the λ, *Y-B*, *R-G* color space is then transformed back to the RGB color space for display.

## 3. Experiments

**Figure 10:**
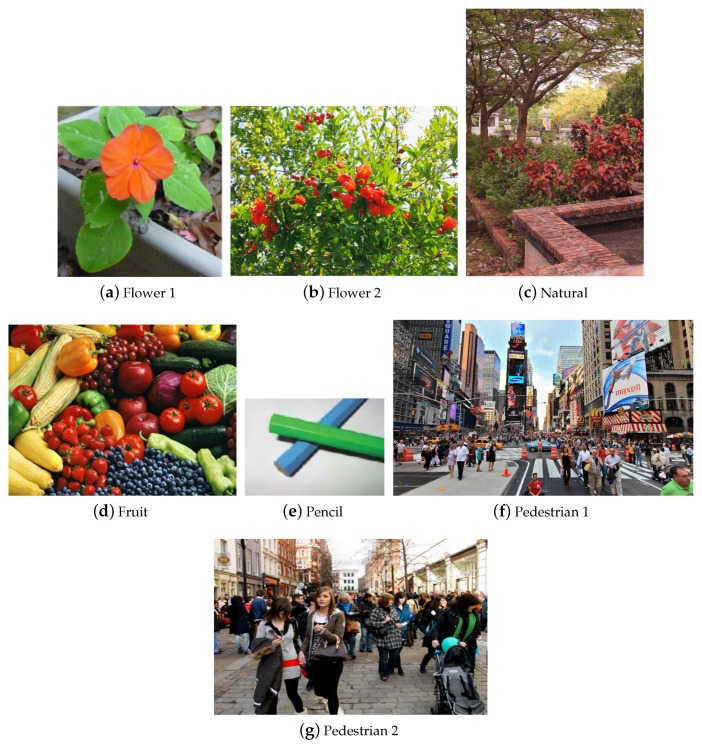
The test images used to evaluate the re-coloring techniques for color vision deficiency.

**Figure 11:**
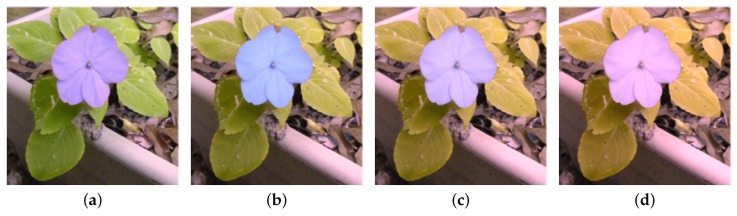
Enhancement sensitive of protanopia color vision deficiency, (**a**) with sensitivity 0.3, (**b**) with sensitivity 0.5, (**c**) with sensitivity 0.7, (**d**) with sensitivity 0.9.

**Figure 12:**
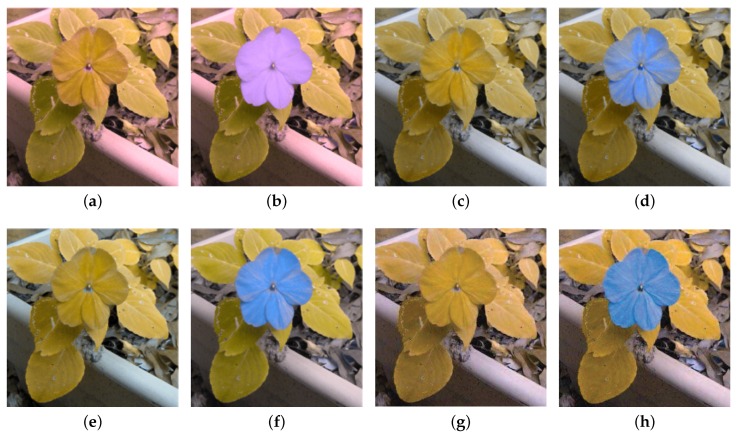
The comparison of deuteranopia simulation of the flower image in Figure 9a. (**a**) Machado’s CVD simulation. (**b**) Our re-coloring technique after Machado’s CVD simulation. (**c**) Brettel’s CVD simulation. (**d**) Kuhn’s re-coloring technique after Brettel’s CVD simulation. (**e**) Rasche’s CVD simulation. (**f**) Rasche’s re-coloring after CVD simulation. (**g**) Huang’s CVD simulation. (**h**) Huang’s re-coloring after CVD simulation.

The proposed method has been tested on natural images including flowers, fruits, pedestrians and landscape, as well as synthetic images such as patterns with pure colors (see Figure 10). The experiments were carried out on both simulation view and human tests. Figure 11 shows the images of protanopia color vision deficiency with different sensitive from 0.3 to 0.9 after our re-coloring technique. For the color vision deficiency view simulation, we compared the results of the proposed approach with the methods presented by Kuhn et al. [37], Rasche et al. [17] and Huang et al. [12]. Figure 12 shows the results of the deuteranopia color vision deficiency simulation and re-coloring using different algorithms. While all methods are able to separate the flower from the leaves, our result is more distinguishable and much closer to original color.

### 3.1. Root Mean Square

We use the root mean square (RMS) value to measure the difference between two images. That is, to evaluate how far between the CVD view simulation and the image processed after our re-coloring algorithm. We calculate the RMS value with k-neighborhood defined by

$$RMS_{i}=\frac{1}{N}\sqrt{\frac{1}{2}\sum_{j=-k}^{k}\left[{\left(a_{i+j}^{r}-a_{i+j}^{t}\right)}^{2}+{\left(b_{i+j}^{r}-b_{i+j}^{t}\right)}^{2}\right]}$$

where $a_{i+j}^{r}$ and $b_{i+j}^{r}$ are $a^{*}b^{*}$ in $L^{*}a^{*}b^{*}$ of the reference image, $a_{i+j}^{t}$ and $b_{i+j}^{t}$ are $a^{*}b^{*}$ in $L^{*}a^{*}b^{*}$ of the target image, and *N* is the number of elements in k-neighbor.

**Figure 13:**
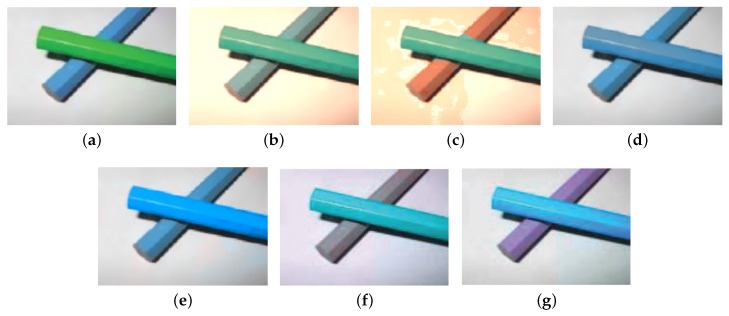
The comparison of tritanopia simulation of the pencil image. (**a**) The original image. (**b**) The CVD simulation using Machado’s method. (**c**) Machado’s CVD simulation on the image processed by the proposed re-coloring technique. (**d**) The CVD simulation using Brettel’s method. (**e**) Brettel’s CVD simulation on the image processed by the Kuhn’s re-coloring technique. (**f**,**g**) CVD simulation and re-coloring using Huang’s approach.

**Figure 14:**
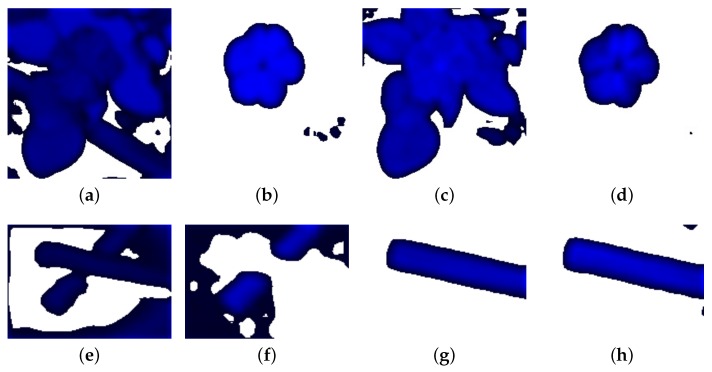
The comparison of RMS values between our method and Kuhn’s method. (**a**) The RMS value between Figure 6a and Figure 12a. (**b**) The RMS value between Figure 12b and Figure 12a. (**c**) The RMS value between Figure 6a and Figure 12c. (**d**) The RMS value between Figure 12d and Figure 12c. (**e**) The RMS value between Figure 13a and Figure 13b. (**f**) The RMS value between Figure 13c and Figure 13b. (**g**) The RMS value between Figure 13a and Figure 13d. (**h**) The RMS value between Figure 13e and Figure 13d.

**Figure 15:**
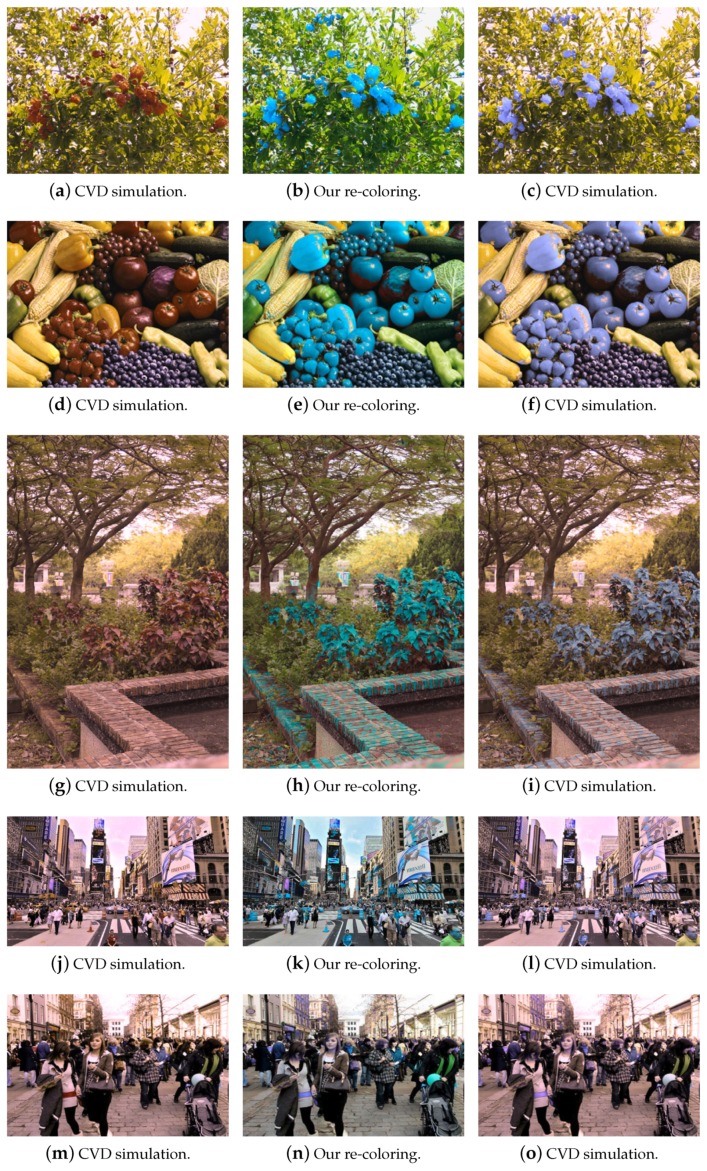
The results of CVD simulation, re-coloring using the proposed technique and CVD simulation on the re-colored images for some test images in Figure 10 (the first two columns) and Figure 15 (the third column).

An example of tritanopia CVD simulation and the re-coloring results is shown in Figure 13. Compared to the results obtained from Kuhn’s and Huang’s methods, our approach provides better contrast between the colors. Figure 14 shows the comparison of the RMS values on several test images using the proposed technique and Kuhn’s method. The higher RMS value is displayed in dark blue, and the lowest value is shown in white. The figures indicate that, although the distributions of our and Kuhn’s results are similar, the RMS values of ours are higher than Kuhn’s, which implies a better separation in colors. Additional results of various types of test images are shown in Figure 15. The results of CVD simulation, re-coloring using the proposed technique and CVD simulation on the re-colored images are shown in the first, second and third column, respectively.

### 3.2. Visual Difference Predictor

**Figure 16:**
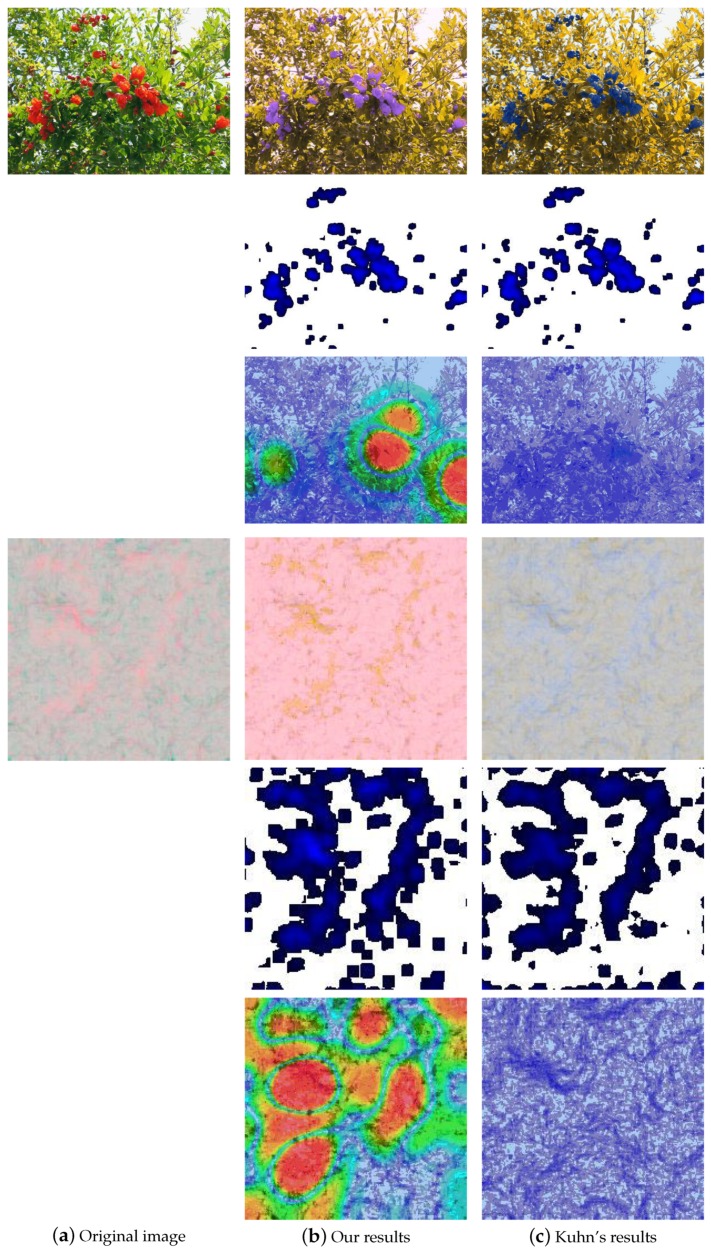
The comparison of CVD simulation results processed using our re-coloring technique and Kuhn’s method. The first and fourth rows are two test images and their CVD simulation results. The second and fifth rows are the visualized RMS values, and HDR-VDP evaluation is shown in the third and sixth rows.

HDR-VDP is a visual metric that compares a pair of images and predicts their visibility (the probability of the differences between the images) and quality (the quality degradation with the respect to the reference image). In this paper, Mantiuk et al.’s HDR-VDP-2 [23] is adopted to evaluate our re-coloring technique and Kuhn’s method. HDR-VDP-2 is a major revision which improves the accuracy of the prediction and changes the metric to predict the visibility (detection/discrimination) and image quality (mean-opinion-score). The new metric also models Long-, Middle-, Short-cone and rod sensitivities for different spectral characteristics of the incoming light. As shown in Figure 16, the first and fourth rows are two test images and their CVD simulation. The images from the left to the right are the original images, CVD simulation results using our re-coloring technique and the results obtained from Kuhn’s method. The second and fifth rows are the RMS values with k=11. The higher value of RMS is displayed in a deeper blue color, and the low value is displayed in a white color. The third and sixth rows are the visibility test results using HDR-VDP-2. The probability of detection map tells us how likely we will notice the difference between two images. Red color denotes the high probability and green color indicates a low probability. Finally, as shown in the second and fifth rows, the distribution of our results and Kuhn’s are almost the same. However, the third and sixth rows indicate that our re-coloring technique is able to provide more distinguishable colors on the CVD simulation results.

### 3.3. Human Subjective Evaluation

The color sensation is commonly considered as a subjective feeling of human beings. In this work, a human test is carried out to evaluate our color enhancement approach. The procedure consists of an image re-coloring stage to produce the CVD-friendly output, and an evaluation stage to analyze the responses collected from the volunteers. In the human test approved by an IRB (institutional review board) [38], people with color vision deficiency were asked to give judgments for the images enhanced by the re-coloring algorithms. We first let the subjects understand the purpose and process of this color test clearly. Three types of color vision deficiencies: protanopia, deuteranopia and tritanopia are considered, and the subjects are classified to groups for testing. Four different approaches, $M_{1},M_{2},M_{3},M_{4}$ are then evaluated as follows.

- $M_{1}$: The input image is converted to the $L^{*}u^{*}v^{*}$ color space, projected to $u^{*}v^{*}$ and equalized the $u^{*}$ and $v^{*}$ coordinates.
- $M_{2}$: The input image is used to simulate the CVD view, and find the (R, G, B) difference between input and simulation images. A matrix is then used to enhance the color difference regions.
- $M_{3}$: The input image is converted to the $L^{*}u^{*}v^{*}$ color space, and rotated to the non-confused color position.
- $M_{4}$: The input image is used to simulate the CVD view, and the distances among the colors are used to obtain the discrepancy. The image is then converted to the λ, *Y-B*, *R-G* color space, and rotated the color difference regions.

We collected 55 valid subjects in the test. The results are tabulated in Table 4. In the table, *i* is the method of different research stages, *j* is the index of the test image, the letters are the feeling level of the pros and cons (denoted by A, B, C, D) for the subjects. The summary indicates the proportion of the method $M_{i}$ for the test image $F_{j}$ chosen by subjects is over 1/3. As shown in Table 4, 83.64% (marked in blue) of 55 subjects selected level A for the method $M_{2}$ and the test image $F_{1}$. The numbers marked in red indicate the proportion of method $M_{i}$ in levels A, B, C, D with higher percentages, and the associated methods are more representative in the level. Thus, each level (A, B, C, D) is represented by the methods: $M_{2}$, $M_{4}$, $M_{3}$, and $M_{1}$. It also shows that the best to the worst for color vision deficiency feeling of the four different methods are given by $M_{2}$, $M_{4}$, $M_{3}$, $M_{1}$.

## 4. Conclusions

In this paper, we present an image enhancement approach to assist colorblind people with a better viewing experience. An image re-coloring method based on eigenvector processing is proposed for robust color separation under color deficiency transformation. It is shown that the eigenvector of color vision deficiency is distorted by an angle in the λ, *Y-B*, *R-G* color space. The proposed method represents clearly subjective image quality and the objective evaluation. Compared to the existing techniques, our results of natural images with CVD simulation work very well in terms of RMS, HDR-VDP-2 and IRB-approved human test. Both the objective comparison with previous works and the subjective evaluation on human tests validate the effectiveness of the proposed technique.

## Figures and Tables

**Table 1:** Cone cells in the human eyes and the response to the light wavelength.

Type	Range	Peak Wavelength
S	400–500 nm	420–440 nm
M	450–630 nm	534–555 nm
L	500–700 nm	564–580 nm

**Table 2:** Approximate percentage occurrences of various types of color vision deficiency [11].

Type	Male (%)	Female (%)
Protanopia	1.0	0.02
Deuteranopia	1.1	0.01
Trianopia	0.002	0.001
Protanomaly	1.0	0.02
Deuteranomaly	4.9	0.38
Tritanomaly	∼0	∼0
**Total**	**8.002**	**0.44**

**Table 3:** Machado’s Simulation Matrices $\Phi_{CVD}$.

Sensitivity	0.6
Protanopia	$\left[\begin{array}{ccc} 0.385 & 0.769 & 0.154\\ 0.101 & 0.830 & 0.070\\ 0.007 & 0.022 & 1.030 \end{array}\right]$
Deuteranopia	$\left[\begin{array}{ccc} 0.499 & 0.675 & 0.174\\ 0.205 & 0.755 & 0.040\\ 0.011 & 0.031 & 0.980 \end{array}\right]$
Tritanopia	$\left[\begin{array}{ccc} 1.105 & 0.047 & 0.058\\ 0.032 & 0.972 & 0.061\\ 0.001 & 0.318 & 0.681 \end{array}\right]$

**Table 4:** The human test on 55 valid subjects with four different methods. The number are shown in percentage. The numbers marked in red indicate the proportion of method $M_{i}$ in levels A, B, C, D with higher percentages, and the associated methods are more representative in the level.

**Level**	**A**				**B**			
	$M_{1}$	$M_{2}$	$M_{3}$	$M_{4}$	$M_{1}$	$M_{2}$	$M_{3}$	$M_{4}$
$F_{1}$	5.45	83.64	5.45	5.45	45.45	9.09	9.09	36.36
$F_{2}$	5.45	90.91	0.00	3.64	3.64	5.45	21.82	69.09
$F_{3}$	1.82	72.73	14.55	10.90	20.00	3.64	25.45	50.91
$F_{4}$	1.82	94.55	1.82	1.82	9.09	1.82	25.45	63.64
e $F_{5}$	1.89	91.67	0.00	8.33	0.00	5.45	20.00	74.55
$F_{6}$	0.00	41.82	12.73	45.45	5.36	8.93	44.64	41.07
$F_{7}$	49.09	9.09	3.64	38.18	16.36	27.27	30.91	25.45
$F_{8}$	14.81	20.37	16.67	48.15	12.37	9.09	50.91	27.27
*Summary*	7.14	64.29	3.57	25.00	11.11	3.70	33.33	51.85
**Level**	**C**				**D**			
	$M_{1}$	$M_{2}$	$M_{3}$	$M_{4}$	$M_{1}$	$M_{2}$	$M_{3}$	$M_{4}$
$F_{1}$	9.09	5.45	54.55	30.91				
$F_{2}$	0.00	0.00	76.36	23.64	90.91	3.64	3.64	1.82
$F_{3}$	9.09	14.55	43.64	32.73	69.09	9.09	16.36	5.45
$F_{4}$	18.18	0.00	49.09	32.73	70.91	3.64	23.64	1.82
$F_{5}$	14.55	1.82	63.64	20.00	88.89	0.00	7.41	3.70
$F_{6}$	21.82	50.91	14.55	12.73	75.47	0.00	24.53	0.00
$F_{7}$	16.36	30.91	32.73	20.00	18.18	32.73	32.73	16.36
$F_{8}$	41.82	16.00	32.00	20.00	31.48	51.85	5.56	11.11
*Summary*	9.68	12.90	58.06	19.35	76.00	12.00	8.00	4.00
